# Safety and one-year follow-up analysis of percutaneous ASD closure at a tertiary care hospital

**DOI:** 10.1016/j.ihj.2025.03.011

**Published:** 2025-03-27

**Authors:** Naga Raghunandan Thota, Kamalakar Kosaraju, John Satish Rudrapogu, Krishna Prasad Nevali, Thirupathi Rao Kondaveeti

**Affiliations:** Department of Cardiology, NRI Medical College, Chinakakani, Andhra Pradesh, 522503, India

**Keywords:** Structural heart defects, Congenital anomalies, Atrial septum, Transcatheter closure, Occluders

## Abstract

**Aim:**

This study was designed to evaluate the safety and effectiveness of the Cocoon Septal Occluder device (Vascular Innovations Co. Nonthaburi, Thailand) for transcatheter closure of isolated secundum type atrial septal defect (ASD) in Indian patients.

**Methods:**

This was a single-center, retrospective, observational study which included patients who underwent transcatheter closure of isolated secundum ASD using the Cocoon Septal Occluder between April 2014 and May 2023. Follow-up assessments up to one-year were conducted through review of hospital medical records, clinic visits, or via telephonic communication with primary care physicians.

**Results:**

A total of 400 patients were included in the study, consisting of 28 paediatric (aged ≤15 years, 8.14 ± 4.41 years) and 372 adult patients (40.83 ± 13.23 years). The mean defect diameter and device size were 16.75 ± 5.85 mm and 20.43 ± 6.24 mm for paediatric patients, and 21.62 ± 6.87 mm and 24.94 ± 7.28 mm for adult patients, respectively. The device was successfully implanted in all paediatric patients, achieving 100 % closure of the defect with no complications, which persisted through one-year follow-up. In the adult cohort, complete ASD closure was achieved in 99.2 % of patients, with two cases of device embolization and one case of device withdrawal. At one-year follow-up, adult patients experienced 0.3 % late device embolization, 0.8 % pericardial effusion/cardiac tamponade, 0.5 % atrioventricular block, and 0.5 % atrial flutter/fibrillation. No cases of endocarditis, haemolysis, nickel allergy, stroke/transient ischemic attack, or migraine were reported in either paediatric or adult patients.

**Conclusion:**

The results demonstrate that Cocoon Septal Occluder is safe and effective in closing isolated secundum ASD during one-year follow-up.

## Graphical abstract

**Figure:**
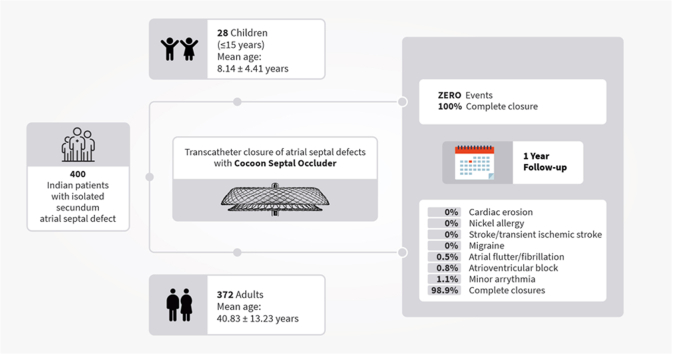

## 1 Introduction

Atrial septal defect (ASD), the second most common congenital cardiac malformation, is characterized by direct communication between atrial chambers. ASD represents around 7–10 % of all congenital heart defects at birth.^1^^,^^2^ Most children and young adults with ASD remain asymptomatic until adulthood, leading to late diagnosis, making it the most frequently diagnosed congenital cardiac defect in adults (25–30 % of new cases).^3^ Among the four types of ASDs—ostium secundum, ostium primum, sinus venosus defect, and coronary sinus defect—the ostium secundum ASD is the most prevalent, accounting for ∼80 % of all ASD cases.^4^

Although King et al,^5^ laid the foundation for transcatheter closure of ASD in 1976, it has evolved substantially in the last three decades. Transcatheter device closure of ostium secundum ASD has become the standard of care in recent years, given its safety and effectiveness relative to surgical closure. Since the inception of the transcatheter approach, multiple ASD closure devices have been designed and have undergone clinical testing. However, the quest for an ideal ASD closure device continues, as complications such as cardiac erosions and nickel allergy persist. The Cocoon Septal Occluder, developed by Vascular Innovations Co. Nonthaburi, Thailand (a Sahajanand Medical Technologies, Ltd. group company), is an ASD closure device featuring a nitinol framework covered with a nanocoating of platinum achieved through nano-fusion technology. This nanocoating enhances radiopacity, ensures excellent biocompatibility, and prevents nickel leaching into the circulation. Published outcome data on transcatheter closure of ASD using Cocoon Septal Occluder are limited in the Indian population.^6^^,^^7^ Therefore, this study aimed to evaluate the safety and effectiveness of the Cocoon Septal Occluder device for transcatheter closure of isolated secundum ASD in Indian patients.

## 2 Methods

### 2.1 Study design and population

This was a single-center, retrospective, observational study conducted at a tertiary care center in India. All patients who underwent transcatheter closure of isolated secundum ASD using the Cocoon Septal Occluder between April 2014 and May 2023 were included in the study. This study was approved by the institutional ethic committee of NRI Medical College and General Hospital, Guntur, Andhra Pradesh, India, approval number (ECR/1160/Inst/AP/2013/RR-07). The written informed consent was obtained from all adult participants and all underaged participant's legal guardian before transcatheter ASD closure.

### 2.2 Study device

**Fig. 1:**
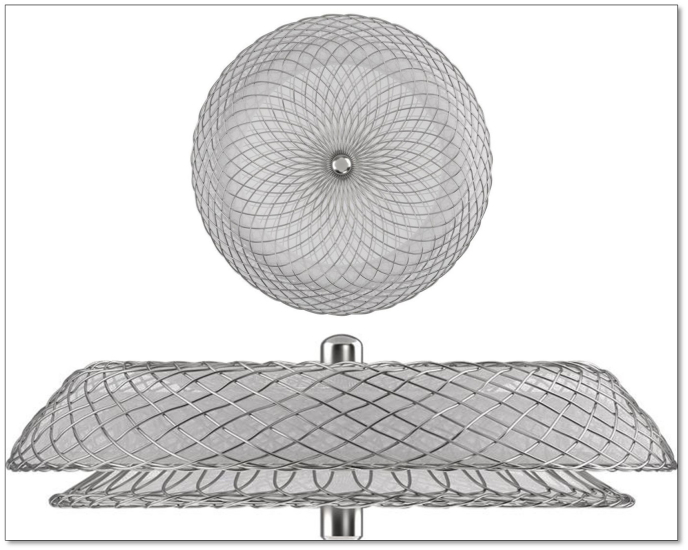
Cocoon septal occluder.

The Cocoon Septal Occluder (Fig. 1) comprises a self-expandable, double-disc device made up of nitinol wire with a Nanoplatinum coating. This coating serves to prevent the leaching of nickel into the bloodstream and guards against the corrosion of the nitinol wire frame during long-term implants. Additionally, it enhances radiopacity, fosters excellent biocompatibility, and smoothens irregularities of the Nitinol wires, resulting in a smooth device surface. The device incorporates multiple polypropylene woven fabrics that expedite faster and complete closure of defects by inducing thrombogenicity. Furthermore, the smaller metal-to-disc ratio of the device makes it magnetic resonance imaging (MRI) compatible and facilitates effortless recapture and repositioning during the procedure. The double discs of the device are connected by a central waist, ranging from 8 mm to 40 mm with a 2 mm increment, ensuring precise adaptation to varying anatomical needs.

### 2.3 Procedure

Before the procedure, all patients duly underwent catheterization of chambers to assess the right ventricle, right atrium, left ventricular end-diastolic pressure (LVEDP) and pulmonary artery pressure. Patients with high LVEDP >12 mm Hg, were not considered immediately for ASD closure due to the high risk of pulmonary oedema and mortality caused by chronic left ventricular dysfunction. They were treated with diuretics and were subsequently considered only if LVEDP reduced to 8 mmHg. Transcatheter closure of the ASD was performed via femoral vein access, predominantly under local anesthesia, with some cases requiring general anesthesia and transesophageal echocardiography (TEE) guidance, alongside fluoroscopic guidance for all cases. Cardiac catheterization was conducted to assess the patient's hemodynamic status. The device size was selected based on the maximum diameter of the ASD as determined on TEE or using a sizing balloon via "stop-flow technique" with echocardiography and fluoroscopy. Typically, a device size 2 mm larger than the balloon occlusive diameter was selected. However, in cases of significant discrepancies in defect size across different views, multiple device sizes were considered, with the most appropriate one ultimately implanted. During the procedure, all the patients were heparinized to achieve an activated clotting time of >200 s at the time of device implantation. Intravenous heparin (100 IU/kg) was administered during the procedure. The device was positioned at the defect using the standard technique, and its position and stability were verified before it was released from the loader system.

### 2.4 Data collection and follow-up

According to the center, TTE or TOE was routinely conducted prior to ASD closure for all patients. Anatomic ASD features were recorded including ASD size (in mm, largest ASD diameter on any view), presence and location of deficient rims defined as <5 mm in length (i.e., anteroinferior, posterosuperior, aortic, posterior, inferior, and superior rims). Catheterization reports were used to collect the type of imaging guidance (TOE, TTE, or intracardiac echography), hemodynamic data (mean pulmonary arterial pressures; pulmonary to systemic output ratio [Qp/Qs ratio]), device size, and potential complications.

As per routine practice of the center, all patients underwent clinical examination, electrocardiography, chest radiography, and TOE before discharge, followed by subsequent assessments up to 12 months. Thus, post-procedural outcomes were obtained through hospital medical records review, while outcomes up to one-year follow-up were collected via hospital medical records review, clinic visits, or telephonic communication with primary care physicians. For patients with device sizes greater than 30 mm, aspirin 75 mg and clopidogrel 75 mg were administered for 3 months, followed by aspirin 75 mg alone for an additional 5 months. For smaller devices, aspirin 75 mg was given for 6 months in all age groups above 10 years. In patients younger than 10 years, aspirin was administered at a dose of 3–5 mg/kg daily for 6 months.

### 2.5 Statistical analysis

Continuous variables are expressed as mean (±SD) and categorical variables as frequency (percentages). All data were analyzed using the R software version 4.3.3 (The R Foundation, Vienna, Austria)

## 3 Results

The study included a total of 400 patients who underwent transcatheter closure of secundum ASD using the Cocoon Septal Occluder. These patients were categorized into two groups: 28 paediatric patients (aged ≤15 years) and 372 adult patients with isolated secundum ASD. The mean age was 8.14 ± 4.41 years for paediatric patients and 40.83 ± 13.23 years for adults, with females comprising 57.1 % and 51.9 % of each group, respectively. The mean weight was 38.07 ± 21.18 kg and 64.47 ± 9.94 kg for paediatric and adult patients, respectively. The mean diameter of defect was 16.75 ± 5.85 mm for paediatric and 21.62 ± 6.87 mm for adult patients, and the mean diameter of Cocoon Septal Occluder device was 20.43 ± 6.24 mm for paediatric and 24.94 ± 7.28 mm for adult patients. Isolated deficiency of the aortic rim was found in overall 166 (41.5 %) patients (*n* = 11, 39.3 % in paediatric patients and *n* = 155, 41.7 % in adult patients) and of the IVC posterior rim in overall 145 (36.3 %) patients. During the procedure, the left upper pulmonary vein was hooked in 380 patients; however, variants were used in 20 patients, with the right upper pulmonary vein accessed in 15 patients and left lower pulmonary vein in five patients. During the procedure, cobra formation was observed in three patients but was successfully resolved by pull back. Furthermore, balloon assisted technique was performed in four patients due to mismatch in the actual size of the rims, the device and echo evaluation. The details of baseline and procedural characteristics are highlighted in Table 1.

**Table 1:** Baseline demographic and procedural details.

Patient Characteristics	Children (1–15 years) *n* = 28	Adult (16–69 years) *n* = 372
Age, years, (mean ± SD)	8.14 ± 4.41	40.83 ± 13.23
Female, n (%)	16 (57.1 %)	193 (51.9 %)
Height, cm (mean ± SD)	96.21 ± 55.47	164.95 ± 8.23
Weight, kg (mean ± SD)	38.07 ± 21.18	64.47 ± 9.94
Procedural Details		
Qp/Qs Ratio, (mean ± SD)	1.68 ± 0.37	1.94 ± 0.43
Pulmonary Hypertension, n (%)		
Systolic, mmHg (mean ± SD)	36.93 ± 5.15	40.38 ± 8.00
Diastolic, mmHg (mean ± SD)	13.74 ± 1.68	13.60 ± 2.05
Size of Defect mm (mean ± SD)	16.75 ± 5.85	21.62 ± 6.87
Aortic Rim, n (%)		
Present	17 (60.7 %)	217 (58.3 %)
Deficient	11 (39.3 %)	155 (41.7 %)
Other Deficient Rims, n (%)		
Superior vena cava	8 (28.6 %)	81 (21.8 %)
Anterior	13 (46.4 %)	148 (39.8 %)
IVC Posterior	7 (25.0 %)	138 (37.1 %)
IVC Flimsy	0 (0.0 %)	5 (1.3 %)
Device Diameter, mm (mean ± SD)	20.43 ± 6.24	24.94 ± 7.28
10 mm	1 (3.6 %)	0 (0.0 %)
12 mm	0 (0.0 %)	10 (2.7 %)
14 mm	4 (14.3 %)	27 (7.3 %)
16 mm	4 (14.3 %)	16 (4.3 %)
18 mm	5 (17.9 %)	20 (5.4 %)
20 mm	3 (10.7 %)	55 (14.8 %)
22 mm	3 (10.7 %)	20 (5.4 %)
24 mm	2 (7.1 %)	41 (11.0 %)
26 mm	3 (10.7 %)	59 (15.9 %)
28 mm	1 (3.6 %)	40 (10.8 %)
30 mm	0 (0.0 %)	9 (2.4 %)
32 mm	0 (0.0 %)	23 (6.2 %)
34 mm	0 (0.0 %)	5 (1.3 %)
36 mm	2 (7.1 %)	11 (3.0 %)
38 mm	0 (0.0 %)	14 (3.8 %)
40 mm	0 (0.0 %)	22 (5.9 %)

Qp/Qs ratio: the ratio between pulmonary (Qp) and systemic flow (Qs).

The Cocoon Septal Occluder device was successfully implanted, achieving complete closure of ASD in all 28 paediatric patients (100 %) and in 369 of 372 adult patients (99.2 %). Among paediatric patients, no cases of device embolization, device withdrawal, perforation, stroke/transient ischemic attack, or complete atrioventricular block were observed. However, two cases of post-procedural vascular bleeding and one case of type I atrioventricular block were reported. In the adult population, procedural complications included two cases of device embolization, one case of device withdrawal, and one case of perforation during implantation. Detailed procedural outcomes for both paediatric and adult patients are presented in Table 2.

**Table 2:** Post-procedural in-hospital outcomes.

In-hospital Outcomes	Children (1–15 years) *n* = 28	Adult (16–69 years) *n* = 372
Complete Closure, n (%)	28 (100.0 %)	369 (99.2 %)
Device Embolization, n (%)	0 (0.0 %)	2 (0.5 %)
Device Withdrawal, n (%)	0 (0.0 %)	1 (0.3 %)
Perforation, n (%)	0 (0.0 %)	1 (0.3 %)
Thrombus Formation, n (%)	0 (0.0 %)	0 (0.0 %)
Vascular Bleeding, n (%)	2 (7.1 %)	8 (2.2 %)
Atrial Arrhythmia, n (%)	0 (0.0 %)	5 (1.3 %)
Stroke/Transient ischaemic attack, n (%)	0 (0.0 %)	0 (0.0 %)
Atrio-ventricular block, n (%)		
Type-I	1 (3.6 %)	7 (1.9 %)
Type-II	0 (0.0 %)	2 (0.5 %)
Complete atrio-ventricular block, n (%)	0 (0.0 %)	0 (0.0 %)

All patients were followed for one-year post-ASD closure with the Cocoon Septal Occluder device (Table 3). At the one-year 2D echocardiographic evaluation, complete closure was observed in all 28 paediatric patients (100 %), with all devices correctly positioned across the atrial septum and no instances of late device embolization, cardiac erosion, perforation, or thrombus formation. In the adult patients, one case (0.3 %) of late device embolization, three cases (0.8 %) of pericardial effusion or cardiac tamponade, three cases (0.5 %) of atrioventricular block, four cases (1.1 %) of minor atrial arrhythmia, and two cases (0.5 %) of atrial flutter/fibrillation were reported during the one-year follow-up. No cases of endocarditis, haemolysis, nickel allergy, stroke/transient ischemic attack, or migraine were reported in either paediatric or adult patients.

**Table 3:** Post-discharge one-year follow-up outcomes.

Outcomes at one-year follow-up	Children (1–15 years) *n* = 28	Adult (16–69 years) *n* = 372
Complete Closure, n (%)	28 (100 %)	368 (98.9 %)
Embolization, n (%)	0 (0.0 %)	1 (0.3 %)
Cardiac Erosion, n (%)	0 (0.0 %)	0 (0.0 %)
Nickel Allergy, n (%)	0 (0.0 %)	0 (0.0 %)
Pericardial Effusion/Cardiac Tamponade, n (%)	0 (0.0 %)	3 (0.8 %)
Endocarditis, n (%)	0 (0.0 %)	0 (0.0 %)
Thrombus Formation, n (%)	0 (0.0 %)	2 (0.5 %)
Haemolysis, n (%)	0 (0.0 %)	0 (0.0 %)
Atrio-ventricular block, n (%)	0 (0.0 %)	3 (0.8 %)
Stroke/Transient ischaemic attack, n (%)	0 (0.0 %)	0 (0.0 %)
Minor Atrial Arrhythmia, n (%)	0 (0.0 %)	4 (1.1 %)
Atrial Flutter/Fibrillation, n (%)	0 (0.0 %)	2 (0.5 %)
Migraine/Headache, n (%)	0 (0.0 %)	0 (0.0 %)

## 4 Discussion

The present study outlines the clinical experience with the Cocoon Septal Occluder for transcatheter closure of isolated secundum ASD in Indian patients. The key findings include – i) successful deployment of the Cocoon Septal Occluder resulted in complete closure of the defect in all 28 paediatric patients (100 %) and in 369 of 372 adult patients (99.2 %); ii) no immediate or long-term complications (up to one-year follow-up) were reported following Cocoon Septal Occluder deployment among pediatric population (age ≤15 years), iii) no cases of cardiac erosion and nickel allergy were noted in either the adult or paediatric populations up to one-year follow-up.

For the past three decades, transcatheter closure of secundum ASD has emerged as the established standard treatment option for both paediatric and adult patients. Despite the array of available devices, this approach is still burdened by complications. The Cocoon Septal Occluder, a new-generation ASD closure device, is comprised of nitinol wires intricately woven into two low-profile, self-expandable discs connected by a waist. These Nitinol wires are coated with a platinum nano-layer using nano fusion technology which prevents nickel leakage into the bloodstream, thus minimizing the risk of nickel allergy. This assertion is supported by the findings of the current study, which revealed no reported cases of nickel allergy following the implantation of the Cocoon Septal Occluder. In a study by Thanopoulos et al,^8^ on 4008 patients undergoing transcatheter ASD closure with the same device, 15 patients tested positive for nickel allergy skin test; nevertheless, no patient experienced nickel related allergic reaction either post-procedure or during follow-up. Additionally, among all previous studies that used Cocoon Septal Occluders, no cases of nickel allergy have been reported with Cocoon Septal Occluders.^6^, ^7^, ^8^, ^9^, ^10^, ^11^

The present study demonstrated a 100 % success rate in Cocoon Septal Occluder deployment among paediatric patients, achieving complete defect closure with no residual shunt, allergic reactions to nickel, or device-related complications during the procedure, all persisting through one-year follow-up. In adult patients, the device achieved a 99.25 % success rate, with immediate complete closure in nearly all cases; however, there were two cases of device embolization and one case of device withdrawal. Device embolization is always a concern in patients undergoing transcatheter closure of the defect; in our study, the rate of early or late embolization was 0.75 % (in three cases), with all three cases occurring in adults requiring larger devices. Previous studies on the Cocoon Septal Occluder report device embolization rates ranging from 0 % to 1 %,^6^^,^^8^, ^9^, ^10^, ^11^ which is consistent with the rates observed with other contemporary ASD closure devices.^12^, ^13^, ^14^, ^15^ However, factors such as defect size, inadequate rims, undersizing of the device, and operator experience may influence the risk of device embolization after implantation.

Cardiac erosion is a rare, but life threating complications associated with transcatheter ASD closure devices. The incidence of cardiac erosion ranges from 0.1 to 0.4 % with available septal occluders.^14^^,^^16^^,^^17^ However, no cases of cardiac erosion have been reported with the Cocoon Septal Occluder in the current study as well as in the previously published studies, including long-term follow-up of up to 48 months.^6^^,^^8^, ^9^, ^10^, ^11^ It is worth noting that while there is still insufficient evidence regarding specific causes for device-related cardiac erosion, it has been recognized that device stiffness appears to be one of the significant factors in the development of cardiac erosions.^8^ The absence of cardiac erosion cases associated with the Cocoon Septal Occluder may be attributed to its inherent softness and smooth surface, facilitated by the nanoplatinum coating, which also serves to eliminate irregularities in the Nitinol wires and reduce friction on adjacent cardiac structures.

The incidence of atrial arrhythmia following transcatheter ASD closure ranges between 1.5 % and 5.2 % in the literature.^16^^,^^18^, ^19^, ^20^ In our study, 1.3 % of adult patients experienced new-onset atrial arrhythmias during the hospital-stay. By one-year follow-up, 1.1 % of adults had minor atrial arrhythmias, while 0.5 % experienced atrial fibrillation/flutter. Following device implantation, nine cases of atrioventricular block (seven Type I and two Type II) occurred during the hospital stay, all of which were successfully resolved with medical management without the need for device removal. By one-year follow-up, only three cases of atrioventricular block were reported, which were also managed conservatively. However, no cases of complete atrioventricular block were reported in the entire cohort of 400 patients during the one-year follow-up. The mean age of our adult population was 40.83 ± 13.23 years, and one study suggests that age over 40 years may be a risk factor for arrhythmia after ASD closure.^21^ Notably, no cases of any form of arrhythmia were reported in the pediatric population (age ≤15 years). These findings align with existing literature and underscore the need for careful monitoring of arrhythmias in adult patients, particularly those over 40 years, following transcatheter ASD closure.

## 5 Study limitations

Firstly, the retrospective and non-randomized nature of the study inherently makes it susceptible to certain biases (recall bias). Secondly, the single-center experience restricts the generalizability of the current findings. Therefore, further studies with relatively longer follow-up periods (>1 year) and involving multiple sites across different geographical locations are required to validate these findings. Furthermore, incorporation of 3D and 4D Echo TEE would have further refined preprocedural evaluation and would have prevented device embolization and other complications.

## 6 Conclusion

The findings of the current study demonstrate the safety and effectiveness of the Cocoon Septal Occluder in closing isolated secundum ASD in both the paediatric (age ≤15 years) and adult populations, with successful deployment and complete closure of the defect, accompanied by very few complications up to one-year follow-up. However, broader generalization of these results requires further studies involving more diverse patient cohorts and longer follow-up periods.

## Institutional and financial support

This research did not receive any specific grant from funding agencies in the public, commercial, or not-for-profit sectors.

## Informed consent

The written informed consent was obtained from all the patients before enrolment.

## Presentation at a meeting

Nil.

## Declaration of competing interest

The authors declare that they have no known competing financial interests or personal relationships that could have appeared to influence the work reported in this paper.
